# Loss of *PALB2* predicts poor prognosis in acute myeloid leukemia and suggests novel therapeutic strategies targeting the DNA repair pathway

**DOI:** 10.1038/s41408-020-00396-x

**Published:** 2021-01-07

**Authors:** Antonella Padella, Maria Chiara Fontana, Giovanni Marconi, Eugenio Fonzi, Elisabetta Petracci, Anna Ferrari, Carmen Baldazzi, Cristina Papayannidis, Andrea Ghelli Luserna Di Rorá, Nicoletta Testoni, Gastone Castellani, Torsten Haferlach, Giovanni Martinelli, Giorgia Simonetti

**Affiliations:** 1 Istituto Scientifico Romagnolo per lo Studio e Cura dei Tumori (IRST) IRCCS, Meldola, Italy; 2 Azienda Ospedaliero-Universitaria di Bologna, via Albertoni 15, Bologna, Italia, Istituto di Ematologia “Seràgnoli”, Dipartimento di Medicina Specialistica, Diagnostica e Sperimentale, Università degli Studi, Bologna, Italia; 3 Dipartimento di Medicina Specialistica, Diagnostica e Sperimentale, Istituto di Ematologia “Seràgnoli”, Università degli Studi, Bologna, Italy; 4 MLL Munich Leukemia Laboratory, Munich, Germany

**Keywords:** Cancer genomics, Acute myeloid leukaemia

Dear Editor,

Acute myeloid leukemia (AML) patients carrying complex karyotype or aneuploidies have a very poor prognosis, with a 5-year overall survival (OS) <20%^1^. We and others have shown that these patients are characterized by high genomic instability, along with defects of DNA damage response (DDR) genes^2,3^.

Partner and localizer of BRCA2 (*PALB2*) is a key player in the homology recombination (HR) pathway, since it functions as a scaffold for the HR complex^4^. *PALB2* monoallelic germline mutations are associated with an increased risk of developing breast, pancreatic, and ovarian cancers, whereas biallelic mutations lead to Fanconi anemia^5^. Genomic lesions, including sequence mutations and copy-number alterations (CNAs) of HR and DDR genes, are rare events in sporadic cancers and no recurrent genomic alterations in these genes have been reported so far in AML.

Here, we studied the genomic alterations of *PALB2* in AML, including its molecular interactions and prognostic relevance.

Single-nucleotide polymorphism (SNP) arrays of 119 adult AML cases and whole-exome sequencing (WES) data of 68 adult AML cases were downloaded from the NGS-PTL project repository. Mutational data from the Beat-AML study (*n* = 531) and acute erythroid leukemia (AEL) cohort (*n* = 159) were retrieved from https://www.cbioportal.org/ and https://pecan.stjude.cloud/, respectively. The GSE23452 SNP array (*n* = 144), the GSE14468 (*n* = 486), and The Cancer Genome Atlas (TCGA, *n* = 200) AML transcriptomic datasets were obtained from Gene Expression Omnibus collection (https://www.ncbi.nlm.nih.gov/gds) and https://cancergenome.nih.gov/, respectively. SNP array data files for the NGS-PTL cohort (*n* = 119) are available through the Gene Expression Omnibus public database (accession no. GSE160982). Detailed dataset information and analysis are described in the Supplemental Material section.

We first looked for *PALB2* sequence variations in publicly available genomic datasets (TCGA, Beat-AML, NGS-PTL, and AEL): one somatic frameshift mutation was identified in one AML patient (NM_024675:exon3:T51Qfs*2, TCGA cohort) and two missense mutations (NM_024675:exon 4:Q460R, NM_024675:exon 4:G165C) were identified in two AEL cases. We then asked whether *PALB2* was affected by CNAs. *PALB2* CN loss was detected in 12/233 patients (5.2%), with a minimal common deleted region of 6.6 kb at the C terminus. This region included exon 12, which encodes for the domain responsible for the interaction with the HR proteins RAD51 and BRCA2 and the DNA repair-related polymerase POLH (Fig. 1A and Supplementary Table 1). *PALB2* deletion was associated with CN loss of genomic regions frequently altered in poor-prognosis AML, including chromosome 5q and 17p13 (*p* < 0.05 and false discovery rate <0.25; Fig. 1B and Supplementary Table 2). AML patients carrying *PALB2* genomic loss were older than wild-type cases (median age of 65 years vs. 59 in the wild-type cohort, *p* = 0.044; Table 1) and were characterized by a poor prognosis (75.0% vs. 37.1% of *PALB2* wild-type cases, *p* = 0.016; Table 1).

**Fig. 1:**
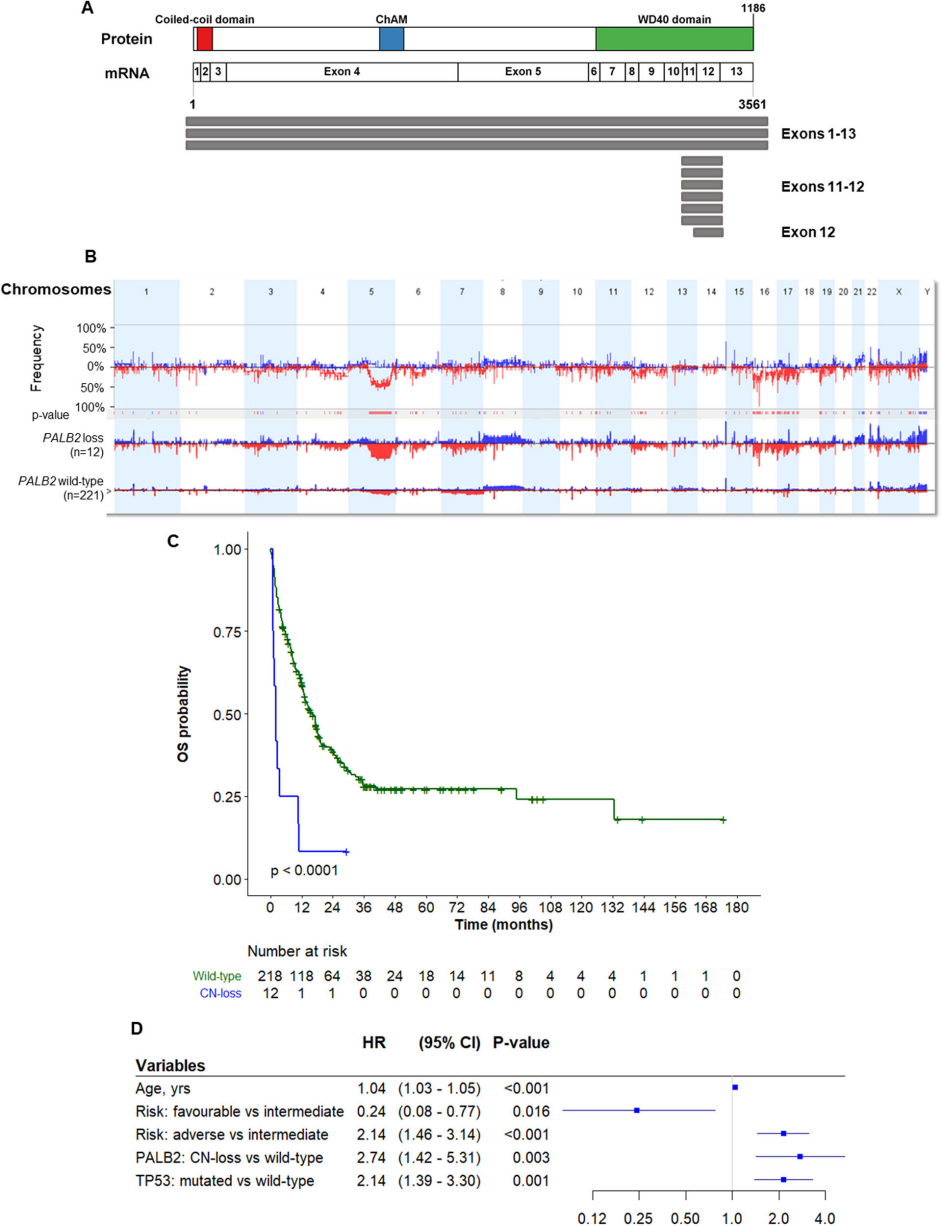
*PALB2* CN loss in AML and its prognostic relevance. **A** Graphical representation of *PALB2* mRNA and protein functional domains. The coiled-coil domain (red) is involved in the interaction with BRCA1; the chromatin-association motif (ChAM, blue) is required for chromatin association and it mediates nucleosome association; the WD40 domain (green) is responsible for the interaction with RAD51, BRCA2, and POLH. Gray bars represent CN loss detected in AML patients. **B** Frequency (%) and the significance of genomic CN changes between *PALB2* loss and wild-type patients. Blue indicates CN gains and red indicates CN loss. Results are summarized for a group of patients (*PALB2* loss vs. *PALB2* wild-type). **C** Kaplan–Meier curve of *PALB2* loss (blue) vs. *PALB2* wild-type (green) AML. **D** Forest plot for the multivariable Cox regression analysis. yrs years, HR hazard ratio, CI confidence interval.

**Table 1:** Comparison of biological and molecular features between *PALB2* loss and *PALB2* wild-type AML cases.

	*PALB2* wt (*n* = 221)	*PALB2* CN loss (*n* = 12)	*P* value
Gender, *n* (%)			1
Female	89 (40.3)	5 (41.7)	
Male	132 (59.7)	7 (58.37)	
Age (years)			0.044
Median [min–max]	59.2 [18.1–88.5]	65.3 [46.6–85.9]	
NA	1	–	
Type of AML, *n* (%)			0.880
De novo	163 (73.8)	10 (83.4)	
Secondary to MN	38 (17.2)	1 (8.3)	
t-AML	20 (9.0)	1 (8.3)	
Risk, *n* (%)			0.016
Favorable	15 (6.8)	1 (8.3)	
Intermediate	124 (56.1)	2 (16.7)	
Adverse	82 (37.1)	9 (75.0)	
*TP53*, *n* (%)			0.001
wt	177 (80.4)	4 (33.3)	
Mutated	43 (19.6)	8 (66.7)	
*NPM1,* *n* (%)			0.370
wt	172 (85.1)	11 (100.0)	
Mutated	30 (14.9)	0 (0.0)	
NA	19	1	
*FLT3*-ITD, *n* (%)			1
wt	182 (87.1)	11 (91.7)	
Mutated	27 (12.9)	1 (8.3)	
NA	12	–	

*wt* Wild type, *CN* copy number, *MN* myeloproliferative neoplasms, *NA* not available, *t-AML* therapy-related AML.

To better understand the phenotype of AML carrying *PALB2* CN loss, we performed pathway analysis of genes significantly affected by CNAs in those patients (compared with *PALB2* wild-type cases, *p* < 0.05). WNT and ERBB2 signaling pathways, cell adhesion, cytokines, and inflammatory response, metabolism, necroptotic process, senescence, and autophagy in cancer were the most relevant pathways enriched of genes affected by CN loss (*p* < 0.01, adj-*p* < 0.25; Supplementary Table 3). By integrating CN and mutational data, we observed an association between *PALB2* deletions and *TP53* genomic alterations: 8/12 *PALB2* loss patients had *TP53* somatic mutations (66.7%), in comparison to 19.6% of *PALB2* wild-type cases (*p* < 0.001; Table 1). Six patients with *PALB2* loss and *TP53* mutations had biallelic inactivation of *TP53* (loss of heterozygosity: *n* = 1; CN loss: *n* = 5), indicating that *TP53* functionality was potentially lost in these cases. To identify additional mutations that cooperated with *PALB2* loss, we integrated CN results with WES data (*n* = 68) of available AML cases from the NGS-PTL cohort^3^ that was enriched for patients having complex karyotype (Supplementary Table 4). In this cohort, 6 out of 68 patients (8.8%) carried *PALB2* CN loss. In addition to *TP53* (*n* = 3, 50.0%), *PALB2* loss patients had mutations in *DNMT3A* (*n* = 2, 33.3%), *BCOR* (*n* = 1), *CDKN2A* (*n* = 1), *FLT3* (*n* = 1), *NPM1* (*n* = 1), *NRAS* (*n* = 1), and *WT1* (*n* = 1, 16.6%; Supplementary Fig. 1).

It has been previously reported that heterozygous *PALB2* genomic alterations, including deletion and truncating or single-nucleotide variants induced a reduction of protein expression^6,7^. Here, we analyzed *PALB2* expression in leukemic cells and its potential association with patients’ biological or molecular features. Data from two independent cohorts revealed that elderly patients expressed lower levels of *PALB2* than younger ones (TCGA: *PALB2* median expression 21.3 vs. 23.3 counts per million, respectively, *p* = 0.007; GSE14468: *PALB2* median expression 213.8 vs. 232.3, respectively, *p* = 0.055; Supplementary Table 5). This result is in line with the above data on AML patients carrying *PALB2* genomic loss. Notably, we observed significant differences in *PALB2* levels among cytogenetic risk classes in the two cohorts (GSE14468, *p* = 0.020 and TCGA, *p* = 0.006). Specifically, patients with favorable prognosis (t(8;21) or inv(16) AML) expressed higher levels of *PALB2*, while intermediate and adverse risk cases had lower expression (normal karyotype, complex karyotypes, *KMT2A*-rearranged AML, and other abnormalities; Supplementary Table 5). This observation also recapitulates the association between *PALB2* genomic loss and high cytogenetic risk. The association between *PALB2* expression and karyotype was not confirmed in the GSE14468 dataset, potentially due to the unbalanced proportion of cases belonging to the different cytogenetic subgroups between the two cohorts.

We then investigated the prognostic role of genetic alterations of *PALB2* in AML. Despite the low number of altered cases, *PALB2* loss patients had a worse prognosis compared to *PALB2* wild-type ones (median OS *PALB2* loss: 2.0 months, 95% confidence intervals (CIs): 1.2–not reached; *PALB2* wild-type: 16.2 months, 95% CIs: 12.9–19.1; *p* < 0.0001; Fig. 1C). Moreover, in a multivariate Cox model *PALB2* loss negatively affected the prognosis of AML patients with a hazard ratio of 2.74 (95% CIs: 1.42–5.31, *p* = 0.003; Fig. 1D) and its effect was not dependent on age, cytogenetic risk, and, notably, on *TP53* mutational status (which were significant predictors in univariate analysis and were in most cases associated with *PALB2* loss). Therefore, *PALB2* might be a novel biomarker of poor prognosis in AML.

*PALB2* is a tumor suppressor affected by mutations and CNAs in patients with either a familiar history of breast, pancreatic, and ovarian cancers^5^, or, rarely, in sporadic solid and hematological tumors (https://www.cbioportal.org/). Notably, *PALB2* participates also in the Fanconi anemia pathway and biallelic germline mutations were associated with the development of AML, among other childhood cancers^4^.

In this study, we report that *PALB2* is rarely mutated at the somatic level in adult AML. However, in a cohort of 233 patients, *PALB2* CN loss was detected in 5.2% of cases and it was associated with *TP53* genomic alterations. Large *PALB2* genomic deletions were also reported in one AML, one myelodysplastic syndrome^8^, and four AEL cases^9^. Remarkably, the co-association of *PALB2* and *TP53* alterations has been previously described in *PALB2*-related breast cancer^10^. Moreover, in our cohort, we detected seven patients with a focal deletion involving exons 11 and 12, which encode for the WD40 domain. Mutations in this domain were reported to disrupt DNA repair activity^6^.

Enrichment analysis of genes within chromosomal regions affected by CN loss and significantly associated with *PALB2* loss pointed to the ERBB2 (HER2) signaling pathway (5/37 enriched pathways, *p* ≤ 0.0114). These data suggest that the phenotype of *PALB2* loss patients might resemble that of HER2-negative breast cancers. Of note, breast cancer patients carrying *PALB2* germline mutations were associated with the HER2-negative phenotype^10^. No familial history of cancer was reported in patients from our AML cohort, suggesting that the detected *PALB2* CN loss might be somatic alterations acquired by the leukemic cells. However, we cannot exclude the germline origin of the deletion, since paired-germline samples were not available.

Few studies described an association between *PALB2* genomic lesions and patients’ outcome so far^11^. We here show a negative impact of *PALB2* genomic alterations on AML patients’ survival with cases carrying *PALB2* loss having shorter OS. This predictive role was independent of other negative prognostic factors (e.g., age, cytogenetic risk, and, notably, *TP53* alterations). Nevertheless, a limitation of our study is the heterogeneity of treatments and patients’ age, pointing out the need for a larger and homogeneous AML cohort to validate the potential of *PALB2* as a biomarker for poor-prognosis AML.

At the therapeutic level, high *PALB2* mRNA levels predicted response to cisplatin–docetaxel in non-small cell lung cancer^12^, while *PALB2*-mutated pancreatic cancers were described to benefit from mitomycin C or PARP inhibition (PARPi) treatment^8,13^. Despite the potential relevance, these studies were characterized by a limited number of patients and the role of *PALB2* alterations in therapy response deserves further investigation. This is particularly important in AML, where functional studies elucidating *PALB2* role in AML cells are needed. PARPi is currently being explored as therapeutic options alone or in combination with chemotherapy, alkylating, or demethylating agents in AML^2^. Different studies reported a deregulated expression of HR genes in AML and preclinical studies showed that t(8;21) and t(15;17) AML are extremely sensitive to PARPi^2,14,15^. Taken together, our data indicate that therapies targeting the HR pathway may become a valuable therapeutic option for the subgroup of AML patients characterized by *PALB2* CN loss and dismal outcome.

## Supplementary information

Supplemental material

Supplemental Figure 1

Supplemental Table 1

Supplemental Table 2

Supplemental Table 3

Supplemental Table 4

Supplemental Table 5
