# Supplementary material for: Loss of PALB2 predicts poor prognosis in acute myeloid leukemia and suggests novel therapeutic strategies targeting the DNA repair pathway
Source: Blood Cancer J. 2021 Jan 7;11(1):7. doi: 10.1038/s41408-020-00396-x (PMC7791026; doi:10.1038/s41408-020-00396-x)
Supplement: Supplementary file 1 — Supplemental material [file 41408_2020_396_MOESM1_ESM.docx]

**Supplemental Materials**

**Sequencing and array datasets**

The following AML datasets were used in this study: NGS-PTL single nucleotide polymorphism (SNP) array cohort^1^ (n=119); GSE23452 SNP array dataset^2^ (n=144); NGS-PTL whole exome sequencing^3^ (WES, n= 68); Beat-AML mutational data^4^ (n=531); acute erythroid leukemia (AEL) data from St. Jude Children's Research Hospital study^5^ (n=159); The Cancer Genome Atlas (TCGA) mutational and RNA-sequencing data^6,7^; GSE14468 gene expression dataset^8^ (n=486).

**Somatic variant detection from WES**

AdapterRemoval (version 1.5.4) was employed to trim reads and remove adapter sequences. Reads were then mapped to the human genome reference GRCh37 (http://ftp.1000genomes.ebi.ac.uk/vol1/ftp/technical/reference/human_g1k_v37.fasta.gz) using BWA-MEM (version 0.7.12). GATK (version 3.4-0) was used for local realignment around insertions/deletions (indels) and base quality scores recalibration. BAM files were analyzed by MuTect (version 1.1.4) and VarScan2 (version 2.3.9) algorithms for somatic variant calling. Variants were annotated with ANNOVAR tool using build version hg19. Oncoprints were generated using R and the package ComplexHeatmap (version 3.6.1 and 2.3.2, respectively).

**Structural variant detection from high-density SNP arrays**

SNP array data was processed by the Affymetrix Genotyping Console and Chromosome Analysis Suite (ChAS version 4.1, Affymetrix, Santa Clara, CA, USA) and analyzed by Nexus Copy Number software (professional version 8.0, Biodiscovery, El Segundo, CA, USA). Events covered by less than 8 probes and smaller than 1 Kb were filtered out. Each segment was compared with the Database of Genomic Variants (DGV, http://dgv.tcag.ca/dgv/app/home), in terms of position and event type. Segments previously reported in healthy subjects were filtered out. For gene/segment analysis, significance and false discovery rate (FDR, q-bound) were calculated as manufacturer’s recommendations (Nexus Copy Number software). Pathway analysis of genes affected by CN alterations was performed on KEGG, Reactome, Wikipath and Gene Ontology Biological processes collections by the Enrichr software and manually reviewed^9,10^. Graphical representation of *PALB2* domains were obtained with Illustrator for Biological Sequences ^11^.

**Transcriptomic data analysis**

RNAseq read counts from the TCGA dataset were transformed into Counts Per Million (CPM) using calcNormFactors (method= ”TMM”) function in edgeR. GSE14468 CEL data were normalized by Transcriptome Analysis Console Software (version 4.0.1) using Robust Multichip Average normalization. Pathway enrichment analysis of genes whose expression correlated with *PALB2* was performed using the package ClueGO^12^ (version 2.5.5) in the Cytoscape environment (version 3.8.0).

**Statistical analyses**

Data were reported as median and minimum-to-maximum values for continuous variables and as natural frequencies and percentages for categorical ones. The Shapiro-Wilk test was used to assess if continuous variables were normally distributed. The association between one continuous and one categorical variable was performed using the Wilcoxon-Mann-Whitney test or the Kruskal Wallis test, as appropriate. The Bonferroni method was used for multiple comparisons, when appropriate. The association between two categorical variables was assessed by means of the Chi-square test or the Fisher’s exact test, as appropriate. Correlation among genes was studied through the Spearman correlation coefficient. The association between variables and OS was investigated using the Kaplan-Meier estimator, the log-rank test for survival curves comparisons and the Cox proportional hazards model (R packages: survival version 3.1-12 and survminer version 0.4.8). Results were reported as point estimates of the median survival or hazard ratio (HR) and corresponding 95% confidence intervals (CIs). Statistical analyses were performed using R statistical language version 3.6.1.

**References**

1. Fontana, M. C. *et al.* Chromothripsis in acute myeloid leukemia: Biological features and impact on survival. *Leukemia* (2017) doi:10.1038/leu.2017.351.

2. Parkin, B. *et al.* Acquired genomic copy number aberrations and survival in adult acute myelogenous leukemia. *Blood* **116**, 4958–4967 (2010).

3. Simonetti, G. *et al.* Aneuploid acute myeloid leukemia exhibits a signature of genomic alterations in the cell cycle and protein degradation machinery. *Cancer* (2018) doi:10.1002/cncr.31837.

4. Tyner, J. W. *et al.* Functional genomic landscape of acute myeloid leukaemia. *Nature* **562**, 526–531 (2018).

5. Iacobucci, I. *et al.* Genomic subtyping and therapeutic targeting of acute erythroleukemia. *Nat. Genet.* **51**, 694–704 (2019).

6. Ley TJ, Miller C, Ding L, Raphael BJ, Mungall AJ, Robertson A, Hoadley K, Triche TJ Jr, Laird PW, Baty JD, Fulton LL, Fulton R, Heath SE, Kalicki-Veizer J, Kandoth C, Klco JM, Koboldt DC, Kanchi KL, Kulkarni S, L. Genomic and Epigenomic Landscapes of Adult De Novo Acute Myeloid Leukemia. *N. Engl. J. Med.* **368**, 2059–2074 (2013).

7. Hoadley, K. A. *et al.* Cell-of-Origin Patterns Dominate the Molecular Classification of 10,000 Tumors from 33 Types of Cancer. *Cell* **173**, 291-304.e6 (2018).

8. Wouters, B. J. *et al.* Double CEBPA mutations, but not single CEBPA mutations, define a subgroup of acute myeloid leukemia with a distinctive gene expression profile that is uniquely associated with a favorable outcome. *Blood* **113**, 3088–3091 (2009).

9. Kuleshov, M. V. *et al.* Enrichr: a comprehensive gene set enrichment analysis web server 2016 update. *Nucleic Acids Res.* (2016) doi:10.1093/nar/gkw377.

10. Chen, E. Y. *et al.* Enrichr: Interactive and collaborative HTML5 gene list enrichment analysis tool. *BMC Bioinformatics* **14**, (2013).

11. Liu, W. *et al.* IBS: An illustrator for the presentation and visualization of biological sequences. *Bioinformatics* **31**, 3359–3361 (2015).

12. Mlecnik, B., Galon, J. & Bindea, G. Comprehensive functional analysis of large lists of genes and proteins. *J. Proteomics* **171**, 2–10 (2018).

**Supplemental Tables**

**Supplemental Table 1.** Genomic details of *PALB2* copy number loss detected by SNP array in 12 AML.

**Supplemental Table 2.** Genomic regions affected by CN changes in patients with *PALB2*-loss when compared to *PALB2* wild-type AML (p<0.05 and q-bound<0.25).

**Supplemental Table 3.** Pathway enrichment analysis of genes significantly affected by CNAs in *PALB2*-loss patients.

**Supplemental Table 4.** Characteristics of the SNP array and WES AML cohorts.

**Supplemental Table 5.** Summary of association studies between *PALB2* expression levels and clinical, biological and molecular features in two public transcriptomic datasets (TCGA and GSE14468).

**Supplemental Figures**

**Supplemental Figure 1. Genomic landscape of *PALB2* CN loss AML.** Oncoprint of leukemia-related genes in *PALB2* CN loss and *PALB2* wild-type AML. Each column represents one patient and each row corresponds to the presence of somatic mutations in defined genes. ITD: internal tandem duplication.
