# Supplementary material for: Loss of PALB2 predicts poor prognosis in acute myeloid leukemia and suggests novel therapeutic strategies targeting the DNA repair pathway
Source: Blood Cancer J. 2021 Jan 7;11(1):7. doi: 10.1038/s41408-020-00396-x (PMC7791026; doi:10.1038/s41408-020-00396-x)

DDR mutated genes in the AML cohort

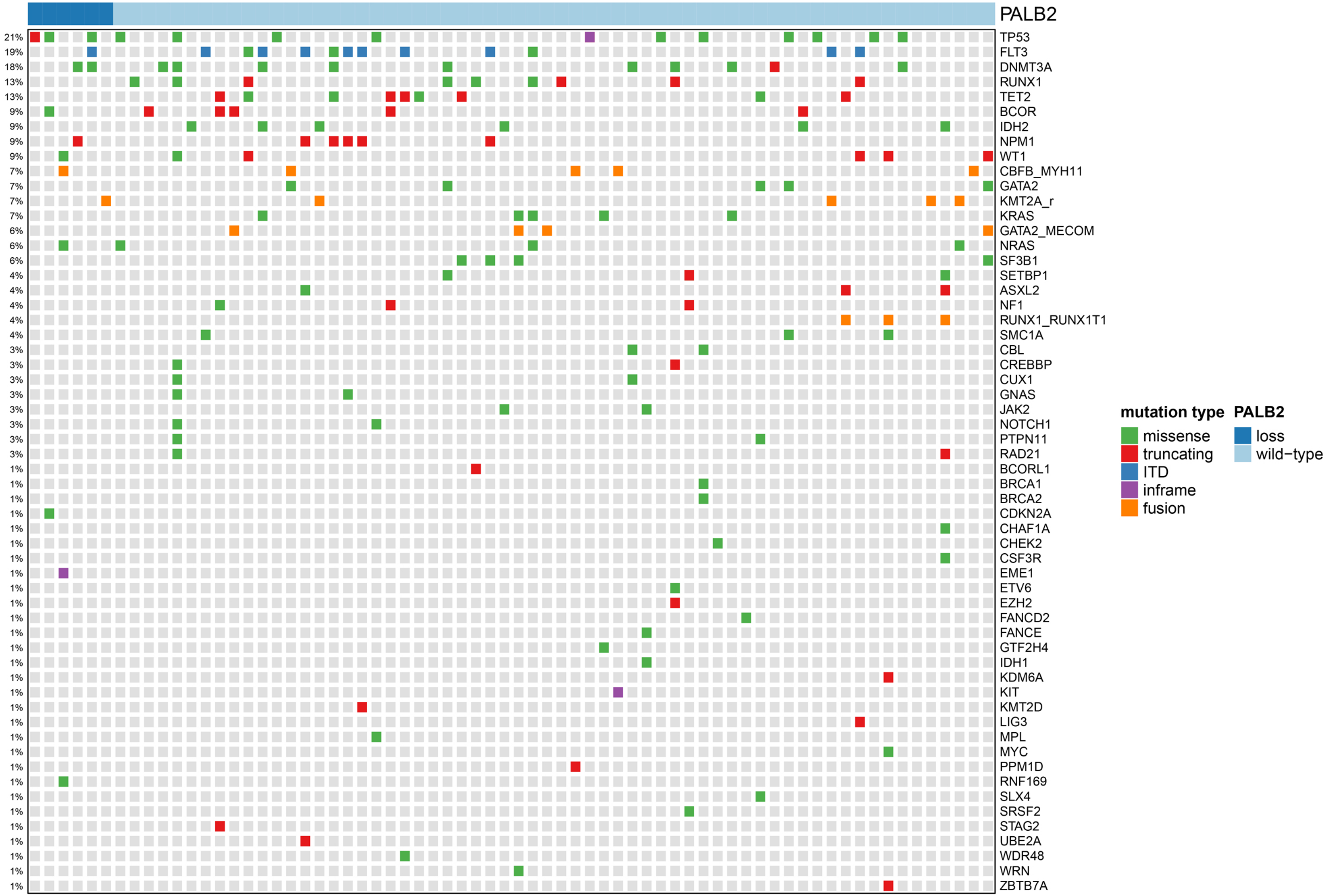

Supplement: Supplementary file 2 — Supplemental Figure 1 [file 41408_2020_396_MOESM2_ESM.pdf]
